# ActinoBase: tools and protocols for researchers working on *Streptomyces* and other filamentous actinobacteria

**DOI:** 10.1099/mgen.0.000824

**Published:** 2022-07-01

**Authors:** Morgan Anne Feeney, Jake Terry Newitt, Emily Addington, Lis Algora-Gallardo, Craig Allan, Lucas Balis, Anna S. Birke, Laia Castaño-Espriu, Louise K. Charkoudian, Rebecca Devine, Damien Gayrard, Jacob Hamilton, Oliver Hennrich, Paul A. Hoskisson, Molly Keith-Baker, Joshua G. Klein, Worarat Kruasuwan, David R. Mark, Yvonne Mast, Rebecca E. McHugh, Thomas C. McLean, Elmira Mohit, John T. Munnoch, Jordan Murray, Katie Noble, Hiroshi Otani, Jonathan Parra, Camila F. Pereira, Louisa Perry, Linamaria Pintor-Escobar, Leighton Pritchard, Samuel M. M. Prudence, Alicia H. Russell, Jana K. Schniete, Ryan F. Seipke, Nelly Sélem-Mojica, Agustina Undabarrena, Kristiina Vind, Gilles P. van Wezel, Barrie Wilkinson, Sarah F. Worsley, Katherine R. Duncan, Lorena T. Fernández-Martínez, Matthew I. Hutchings

**Affiliations:** ^1^​ University of Strathclyde, Strathclyde Institute of Pharmacy and Biomedical Sciences, Glasgow, G4 0RE, UK; ^2^​ Department of Molecular Microbiology, John Innes Centre, Norwich, NR4 7UH, UK; ^3^​ Swansea University Institute of Life Science, College of Medicine, Swansea, Wales, UK; ^4^​ Haverford College, Department of Chemistry, USA; ^5^​ Leibniz Institute DSMZ - German Collection of Microorganisms and Cell Cultures GmbH Inhoffenstraße 7B, 38124 Braunschweig, Germany; ^6^​ Division of Bioinformatics and Data Management for Research, Research Group and Research Network Division, Research Department, Faculty of Medicine Siriraj Hospital, Mahidol University, Bangkok, Thailand; ^7^​ Department of Physics, SUPA, University of Strathclyde, Glasgow, G4 0NG, UK; ^8^​ US Department of Energy Joint Genome Institute, Lawrence Berkeley National Laboratory, Berkeley, CA 94720, USA; ^9^​ Lawrence Berkeley National Laboratory, Environmental Genomics and Systems Biology Division, Berkeley, CA 94720, USA; ^10^​ Department of Insect Symbiosis, Max Planck Institute for Chemical Ecology, Hans-Knöll-Straße 8, 07745 Jena, Germany; ^11^​ Biology Department, Edge Hill University, St Helens Road, Ormskirk, L39 4QP, UK; ^12^​ School of Biological and Behavioral Sciences, Queen Mary University of London, Mile End Road, London, E1 4NS, UK; ^13^​ Colorifix Ltd, Norwich Research Park, Norwich, UK; ^14^​ Astbury Centre for Structural Molecular Biology, University of Leeds, Leeds, LS2 9JT, UK; ^15^​ Faculty of Biological Sciences, University of Leeds, Leeds, LS2 9JT, UK; ^16^​ Universidad Nacional Autónoma de México, Centro de Ciencias Matemáticas, en Morelia, Michoacán, Mexico; ^17^​ Departamento de Química & Centro de Biotecnología Daniel Alkalay Lowitt, Universidad Técnica Federico Santa María, Laboratorio de Microbiología Molecular y Biotecnología Ambiental, Valparaíso, 2340000, Chile; ^18^​ Host-Microbe Interactomics Group, Wageningen University, 6708 WD Wageningen, The Netherlands; ^19^​ Microbial Biotechnology, Institute of Biology, Leiden University, Rapenburg, The Netherlands; ^20^​ School of Biological Sciences, University of East Anglia, Norwich Research Park, Norwich, NR4 7TJ, UK

**Keywords:** actinobacteria, antibiotics, BGCs, CRISPR, specialised metabolites, *Streptomyces*

## Abstract

Actinobacteria is an ancient phylum of Gram-positive bacteria with a characteristic high GC content to their DNA. The ActinoBase Wiki is focused on the filamentous actinobacteria, such as *Streptomyces* species, and the techniques and growth conditions used to study them. These organisms are studied because of their complex developmental life cycles and diverse specialised metabolism which produces many of the antibiotics currently used in the clinic. ActinoBase is a community effort that provides valuable and freely accessible resources, including protocols and practical information about filamentous actinobacteria. It is aimed at enabling knowledge exchange between members of the international research community working with these fascinating bacteria. ActinoBase is an anchor platform that underpins worldwide efforts to understand the ecology, biology and metabolic potential of these organisms. There are two key differences that set ActinoBase apart from other Wiki-based platforms: [1] ActinoBase is specifically aimed at researchers working on filamentous actinobacteria and is tailored to help users overcome challenges working with these bacteria and [2] it provides a freely accessible resource with global networking opportunities for researchers with a broad range of experience in this field.

## Significance as a BioResource to the community

This article describes ActinoBase.org, a community-led website which provides free protocols and other research tools alongside general information and networking for students and scientists interested in studying filamentous actinobacteria. These bacteria are important to humans because they make around two thirds of all known antibiotics including the majority of those used in medicine and agriculture. They are also models for studying complex development and cell division in bacteria. ActinoBase is designed to enable researchers to meet each other and share information and know-how and, ultimately, to make it easier for people to work with these bacteria. The ultimate aim is to enable better understanding of their life cycles, their ecology and their natural products and to use this knowledge to drive the discovery of new molecules that could be useful for a range of applications, including treating disease.

## Data Summary

The authors confirm all supporting data, code and protocols have been provided within the article or through supplementary data files.

## A brief introduction to the Actinobacteria

The phylum Actinobacteria (recently proposed to be renamed Actinomycetota [1]) is a group of high G+C bacteria that includes multicellular filamentous bacteria such as *Streptomyces* species. These filamentous bacteria have attracted much interest because of their complex developmental life cycles, in which development and differentiation are often correlated with the production of specialised metabolites (SMs) [2–4]. The *Streptomyces* life cycle begins with spore germination and outgrowth into a saprophytic, substrate mycelium which grows by hyphal tip extension and branches through the soil, releasing exoenzymes to break down complex organic polymers such as chitin, lignin and cellulose [5]. Stress signals, including nutrient starvation, trigger the formation of aerial hyphae, reproductive structures which undergo rapid DNA replication and cell division to form chains of unigenomic spores. These spores can be dispersed by insects and by motile bacteria or lay dormant in the soil until conditions improve and then germinate to start another life cycle. They are not just free-living soil bacteria however, they also form stable interactions with invertebrates and plants and a few species are plant pathogens [6–8]. Considerable progress has been made to understand *Streptomyces* development over the last 50 years, including the identification of key regulators named Bld and Whi proteins [9, 10]. The Bld proteins are required for aerial hyphae formation, and *bld* mutants have a bald appearance which is characteristic of colonies that lack aerial hyphae. The master regulator BldD governs the entry into sporulation and is controlled by the secondary messenger cyclic-diGMP (c-di-GMP), whereby c-di-GMP activates BldD and causes it to repress all of its target genes and block aerial hyphae formation [11–13]. The Whi proteins are required for sporulation and are so-called because *whi* mutants lack the characteristic spore pigment (grey in the model organism *S. coelicolor* and green in *S. venezuelae *) such that colonies appear white on agar plates. The sigma factor WhiG is also controlled by c-di-GMP [14], which binds to its anti-sigma factor RsiG and makes it repress WhiG to prevent the expression of late stage sporulation genes. Thus, high c-di-GMP levels repress aerial hyphae formation and sporulation to maintain active, vegetative growth, while low c-di-GMP levels trigger differentiation, but the signals and signal transduction systems which control cellular levels of c-di-GMP in *Streptomyces* species are not yet known [15].

Thus, *Streptomyces* species are excellent models for studying complex bacterial differentiation but they are also of great value to humans because they encode many bioactive SMs. These SMs are also known as natural products and they have a variety of applications in human medicine as antibiotics (e.g. chloramphenicol, daptomycin, kanamycin, streptomycin, teicoplanin), anticancer (e.g. bleomycin, doxorubicin), immunosuppressant (e.g. rapamycin, tacrolimus), and antiparasitic drugs (e.g. ivermectin, valinomycin). They are also used in agriculture as insecticides and herbicides. ActinoBase.org was conceived and developed as a community portal where scientists working with filamentous actinobacteria can share expertise, knowledge, and protocols. The phylum also includes pathogens such as *Mycobacterium tuberculosis* and *Corynebacterium diphtheriae* as well as many non-pathogenic genera and species that are outside the scope of this work.

Within the filamentous actinobacteria, *Streptomyces* has been the most studied genus, largely because its members are the most prolific producers of SMs. Between 1945 and 1978, 55 % of all the antibiotics discovered came from the genus *Streptomyces* [4]. The genes encoding SM biosynthetic pathways are typically co-localised on the genome, in what are known as biosynthetic gene clusters (BGCs). Of the 1926 BGCs experimentally linked to SMs, 640 of these are in *Streptomyces* genomes (MIBiG 2.0). *Streptomyces* bacteria are ubiquitous in soils and also found in aquatic niches, with more than 600 known species and thousands of strain variants (https://www.bacterio.net/genus/streptomyces). The first *Streptomyces* genomes to be sequenced and published were those of *S. coelicolor* A3(2) and *S. avermitilis* [16] and examination of the *S. coelicolor* genome revealed that it encoded the potential biosynthesis of 22 SMs, 18 more than were known to be produced under standard laboratory culture conditions [17]. Similar discoveries have now been made for many genome sequenced *Streptomyces* species and this has led to the term silent BGCs, which describes gene clusters that are not expressed, and cryptic BGCs, which is a term used to describe gene clusters whose end products are not known [18]. Since the *S. coelicolor* genome was published, more than 1600 *Streptomyces* genomes have been deposited in public databases and they typically contain between 20 and 60 SM BGCs per genome [19]. Since less than 25 % of these SMs are known to be produced under standard laboratory culture conditions, this strain collection represents a massively under sampled source of new chemistry [20]. A major focus of researchers around the world is to understand how to activate the production of cryptic SMs and to screen these products for bioactivity [21, 22].

Many *Streptomyces* species can be readily isolated from any soil, but less well studied genera of filamentous actinobacteria such as *Amycolatopsis *, *Pseudonocardia *, *Saccharopolyspora *, and *Salinispora* are often described as rare because they are recovered less frequently. *Pseudonocardia* species, for example, are slow-growing and difficult to culture, and have been most commonly found living in a mutually-beneficial symbiosis with attine ants in South and Central America but they have also been isolated from soil and deep-sea marine sediments, suggesting a combination of free-living and symbiotic species in this genus [23–28]. The marine environment is the natural niche of *Salinispora* species, which have been described as the ‘* Streptomyces* of the sea’ because they are also prolific producers of SMs including molecules currently undergoing clinical trials [24]. Identifying new species from underexplored niches, and understanding how to elicit the production of cryptic SMs in these bacteria, offers an opportunity to discover novel chemical compounds, some of which could be used to treat drug resistant bacterial and fungal infections and other diseases. Isolation and genome sequencing of new species from symbiotic niches [26, 29] as well as studies of actinobacteria interacting with other microbes or higher eukaryotes has already led to the identification of novel SMs, including through the activation of cryptic BGCs [30, 31]. For recent reviews on the ecology of actinomycetes we recommend the following articles: [6, 7, 32]. SM biosynthetic pathways have evolved over several billions of years to target essential cellular processes, and it is generally recognised that their discovery remains the best strategy to obtain novel bioactive chemistry, including the next generation of antimicrobial agents.

## A brief history of Actinomycete research

The first member of the phylum Actinobacteria to be discovered was the causative agent of leprosy, *Mycobacterium leprae *, closely followed by *Mycobacterium tuberculosis *, the causative agent of tuberculosis. In between these discoveries was the first description of a filamentous species, which was initially called *Streptothrix* (literally ‘twisted hair’) and later renamed *Streptomyces* (‘twisted fungus’) by Selman Waksman and Arthur Henrici. Following the discovery of the first natural product antibiotic penicillin, Waksman switched his efforts to discovering antibiotics from *Streptomyces* bacteria and, with his graduate student Albert Schatz, discovered streptomycin which was the first antibiotic to be used to successfully treat tuberculosis. This triggered a golden age of antibiotic discovery which peaked in 1955; during this time, most of the antibiotics that are still in use today were discovered [4]. This was a global effort, with researchers in both industry and academia, in the US, UK, Japan, and Europe contributing extensively [33–36]. It also generated interest in the biology of *Streptomyces* bacteria, particularly by a graduate student at Cambridge called David Hopwood who, with an electron microscopist called Audrey Glauret, proved definitively that these microorganisms were bacteria and not fungi [37]. It was Hopwood and colleagues at the John Innes Centre that published the *Practical Streptomyces Genetics* manual in 1985 and updated it in 2000 [38]. This manual is an invaluable resource, particularly for protocols and plasmid maps, but it is neither easily accessible for many members of our community, nor very practical to expand and update given the rapid pace of scientific advances. Thus, the ActinoBase Wiki (actinobase.org) was born out of a desire to connect research groups and facilitate knowledge exchange within the community.

ActinoBase aims to *i*) preserve the knowledge, protocols, and techniques made primarily accessible by the content of the original *Practical Streptomyces Genetics* manual; *ii*) extend its remit to cover techniques developed since 2000, including up-to-date ‘omics-based and computational tools; *iii*) engage the community on the platform; and *iv*) make it freely accessible to anyone around the globe. It features a world map (http://actinobase.org/index.php/Groups) which shows the updated locations of research groups in the ActinoBase community, along with their contact information.

Over the last 60 years, the community has expanded significantly but still remains relatively small when compared with research communities studying *Bacillus* spp. or *Escherichia coli *. This is likely because filamentous actinobacteria are more challenging to culture and genetically manipulate. However, advances in genome sequencing and analysis tools, as well as expanded genetic toolkits, have opened up new opportunities and reduced or removed some of these barriers. For instance, the development of tools such as CRISPR-Cas9 genome editing for *Streptomyces* species [39–42] alongside useful (albeit not actinobacterial-specific) bioinformatic tools, such as antiSMASH [43, 44], autoMLST [45], and ClusterFinder [46] have proven to be major contributions to this scientific field. The community driven ActinoBase Wiki aims to draw on the experience of researchers worldwide and bring it together into a free and easy-to-use online interface. A key goal is to make the Wiki accessible to the broader microbiology community and enable more scientists to join this field.

## The structure of ActinoBase.org

**Fig. 1.**
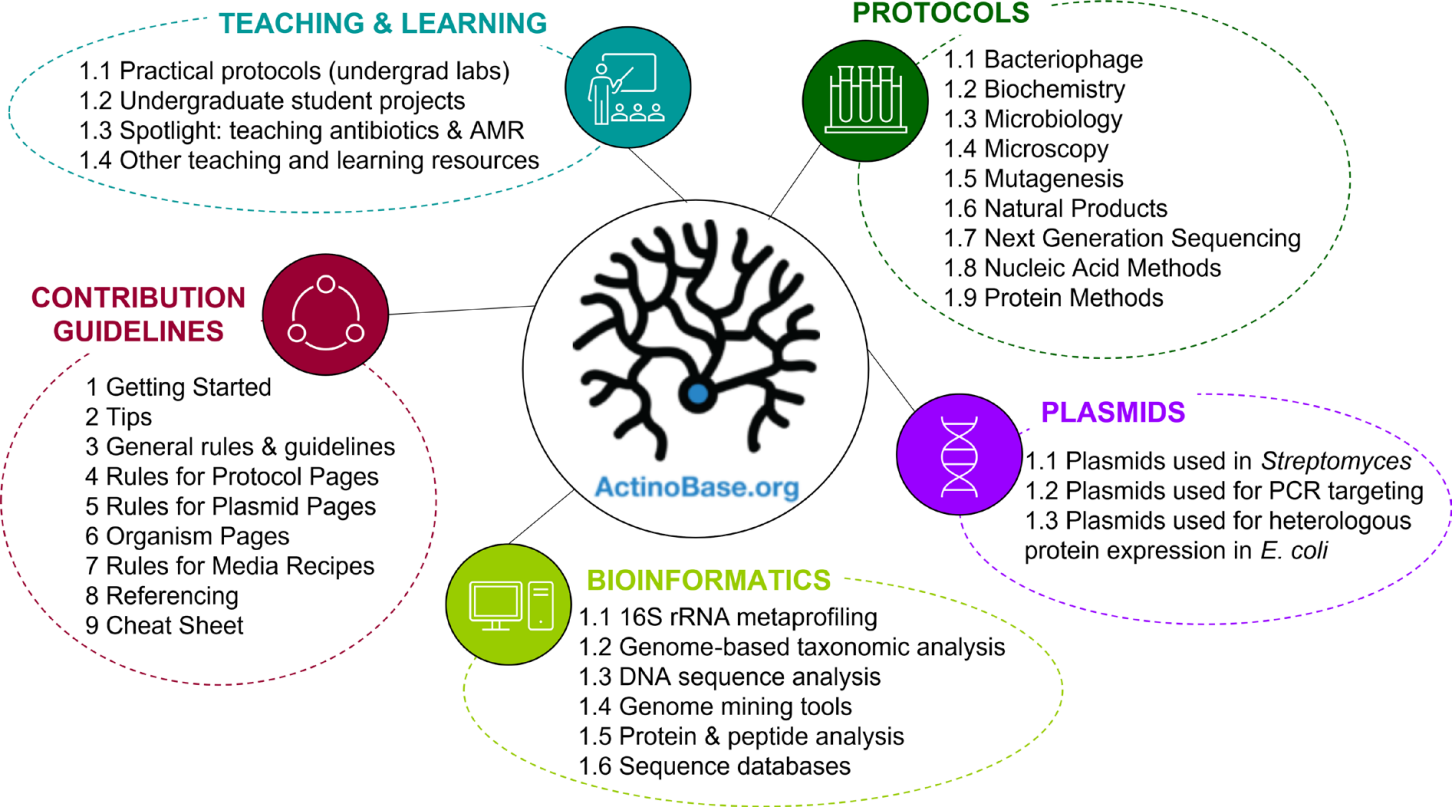
Structure of the ActinoBase Wiki (as of September 2021), with the structure of selected categories (Protocols, Plasmids, Bioinformatics, Teaching and Learning, Contribution Guidelines) shown. The main navigation menu (represented by the ActinoBase logo) is a sidebar accessible from any page in the database.

The structure of ActinoBase is represented in Fig. 1. A navigation menu is found in the left sidebar and offers a core set of links to explore the Wiki content; users can browse through protocols alphabetically or by category, access teaching and learning resources, plasmid information, find details on how to contribute to the Wiki, and access other useful information, by clicking on the link to the appropriate category. Alternatively, the search bar at the top of the page allows users to search directly for a desired page or topic.

Flexibility is a key aspect of the ActinoBase platform. This addresses several of the challenges involved in working with filamentous actinobacteria. For example, irreproducible growth of study organisms can be solved by adjusting protocols on the go, keeping the community up-to-date with the most recent advances and facilitating the spread of specialised know-how. As such, ActinoBase is a dynamic, editable Wiki, where users can share and record their strain-specific experiences, which ultimately benefits the scientific community [47].

Preserving and sharing the knowledge and protocols found in *Practical Streptomyces Genetics* is also an important priority of ActinoBase as the Wiki format means this knowledge is not static, but can be regularly revised and updated and the ActinoBase community can be asked for advice, which happens frequently via the @ActinoBase Twitter feed. Furthermore, ActinoBase links numerous *in silico* analysis tools which did not exist when the latest version of the handbook was published in 2000. Examples of these include the *Streptomyces* Annotation Server - StrepDB (http://strepdb.streptomyces.org.uk/) and comparative genomic resources such as BiG-SCAPE [48] and BiG-SliCE [49], that process the huge amount of data produced by genome mining tools such as antiSMASH [43, 44]. Databases such as antiSMASH-DB [50], BIG-FAM [51] and MiBiG [52] organise years of antiSMASH results, while BiG-SCAPE [48] and BiG-SLiCE [49, 53] software classify BGCs into families that encode potentially similar SMs. Thereby, gene cluster networking analyses can help to prioritise strains in respect to the abundance of unique BGCs which may have the potential to code for novel SMs. Another resource for researchers, is the Multi-Omics Research Factory (MORF-DB, https://www.morf-db.org/projects/Microbiology/ActinoBase). MORF-DB is a genome viewer that provides not only an interactive view of the genome, but also highlights the BGCs identified in the genomes by antiSMASH. Eleven actinobacterial genomes are currently available, ranging through a selection of *Propionibacterium, Pseudonocardia, Salinispora,* and *Streptomyces* species.

ActinoBase also covers ‘wet lab’ advances such as genome manipulation using CRISPR-Cas9, CRISPR-dCas9, and CRISPR-BEST [39–41] along with other methods to generate clean BGC deletions in strains that are difficult to manipulate, such as those which employ the enzymatic function of the meganuclease I-SceI [54]. In addition, ActinoBase includes information on the use of next-generation sequencing (NGS) for applications such as ChIP- and RNA-seq and tools such as cappable RNA-seq which can be used to map transcription start sites [55–57]. Similarly, ActinoBase serves as a hub to explore bacteriophage derived tools for studying *Streptomyces* by linking protocols and external databases. There has been a resurgent interest in phage research over the last decade, ActinoBase is able to stay relevant by drawing on the expertise of its ever-expanding community of users, and by utilising the inherently collaborative nature of the Wiki format. The ‘Bacteriophages’ section is home to routine protocols for the isolation and preparation of bacteriophage, in addition to more advanced genetic techniques such as SV1 phage transduction. Together, these techniques are complemented by pages that detail site-specific phage recombinase targets and external resources such as ‘The Actinobacteriophage Database’. Metabolomics techniques [58] and recent efforts to integrate them with genomic data [59–64] are also absent from *Practical Streptomyces Genetics* and this is an area we plan to incorporate into ActinoBase. Many of these sections are still a work in progress or have yet to be written. We encourage readers to contribute to ActinoBase by expanding the content and uploading relevant resources. An account can be made by contacting the corresponding authors of this manuscript.

Finally, ActinoBase is a universally accessible hub for the community to meet virtually, to share knowledge, and to collaborate. The major meetings in the field have historically been the International Symposium on the Biology of Actinomycetes (ISBA) meetings and the International Symposium on the Genetics of Industrial Microorganisms (GIM), which both go back to the 1960s, and are organised once every 3 years, in different continents. In the pre-digital age, these two meetings were the corner stones of the field. ISBA focuses on fundamental research, while GIM is broader and more applied, including not only actinobacteria but also other industrially used microorganisms.

The global COVID-19 (C19) pandemic has forced the postponement of many conferences, including ISBA, and instead we have seen the introduction of online alternatives. This highlights the importance of active collaboration and community engagement via other means [65]. The restrictions caused by the C19 pandemic, led to the birth of another ActinoBase initiative to share knowledge and information: the eSeminars (http://actinobase.org/index.php/The_ActinoBase_e-Seminar) delivered (and recorded) via the ActinoBase YouTube channel (https://www.youtube.com/channel/UCl-gE1rlZPLfBo7MXuD1DNA). This was promoted to the community via personal networks, the ActinoBase Twitter (@ActinoBase) and the Wiki itself. The impact of such activity in terms of the community is detailed below.

## Functionality

Another advantage of the ActinoBase Wiki over a printed book, which presents information linearly, is that it is easier to connect different pages or link to external resources. This helps the user to better visualise the experimental workflow, e.g. a researcher planning to use intergeneric conjugation to transfer a plasmid from *E. coli* to a recipient *Streptomyces* strain will find the protocol links to all the growth media required for this experiment and a page detailing antibiotic stock and working concentrations. Moreover, the plasmid page contains a map of the plasmid, its sequence, which can be downloaded for further analysis or processing, and a link to the original paper detailing the construction of the plasmid.

ActinoBase.org also provides teaching and learning resources, including a spotlight section about antibiotics and AMR. There is even a section that shows how streptomycetes can be used for creating agar art, taking advantage of the many pigmented natural products made by some of these species [66]. Further to this, there are spaces for researchers to pos jobs and opportunities, making them accessible to the wider community. Links to the eSeminars that have been hosted by ActinoBase and commonly used resources or suppliers are also provided. Finally, there is a section with contribution guidelines which has information for current and new Wiki users who wish to contribute to ActinoBase. New users who would like to create an account are provided with the contact details for the site administrators who are able to set up new accounts. There are also a number of tips to guide a new user in learning the wikitext syntax required to create or edit content, as well as detailed guides to the structure and expected content for new pages (e.g. what is required for a protocol, plasmid, or media recipe page). As a community resource, ActinoBase makes it easy for anyone to upload new content, therefore promoting inclusivity and diverse team participation.

## Applications

ActinoBase is useful for researchers and students: in addition to offering a time saving resource for anyone who needs a quick protocol, we have also designed the Wiki to act as a starting point for new researchers where they can familiarise themselves with the fundamentals. The resource is not limited to researchers however; it is also useful to anyone that wants to learn more about filamentous actinobacteria. ActinoBase is designed to be a hub for numerous interweaving disciplines within the field, which is complementary to the collaborative nature of modern research. Therefore, ActinoBase aims to assist researchers at every step of their scientific investigation. The vision for ActinoBase is to provide a go-to resource for useful information like media recipes and protocols and is positioned as a streamlined portal for accessing external resources, such as databases and bioinformatic tools.

**Fig. 2.**
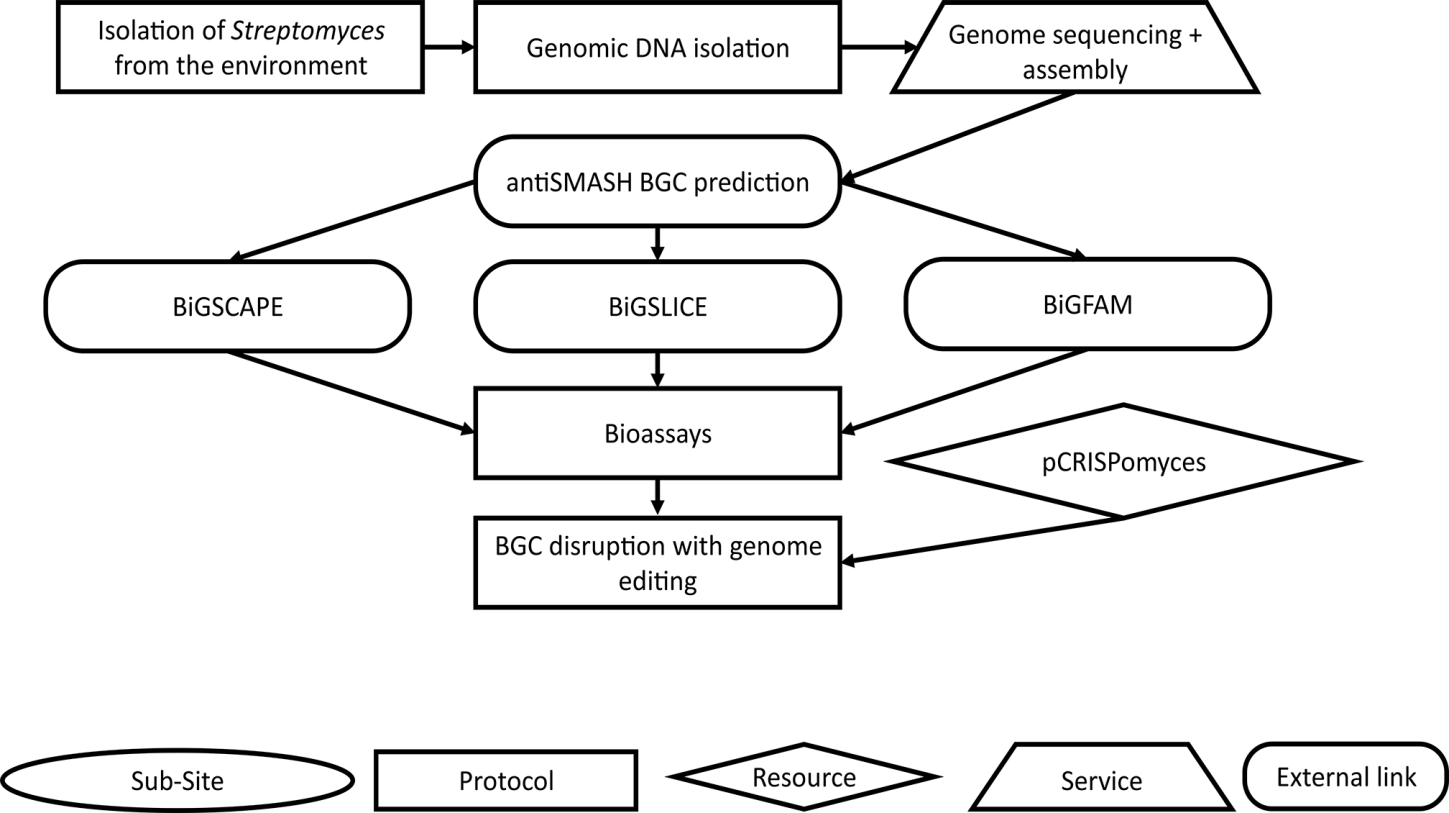
An example workflow for genome mining with *Streptomyces *.

Consider the following case study as an example of how ActinoBase supports research in the biotechnology field (Fig. 2). A researcher has isolated filamentous actinobacteria from the environment and aims to investigate their genetic potential for the production of SMs, by using protocols from ActinoBase. Unlike other bacteria, the extraction of the genomic DNA from these actinobacteria can be challenging; however, the Wiki provides a detailed protocol which helps the researcher extract the DNA successfully (adapted by Jake Newitt from Practical *Streptomyces* genetics [38]). This DNA can be sent away for genome sequencing and assembly. ActinoBase then serves as a portal for bioinformatic resources, which help the researcher to analyse the assembled genome. The Wiki links the researcher to genome mining tools such as antiSMASH [43, 44], which is a BGC prediction tool that can assist in discovering BGCs that may encode bioactive SMs. Following this, BiG-SCAPE [48], BiG-SliCE [53] and BiG-FAM [51] can be used to search for the occurrence of closely related BGCs across other taxonomic lineages, allowing the identification of unique features. Strains with unique BGCs can then be prioritised for further investigation, including bioassays of wild-type versus BGC mutant strains to see if they are active or silent, and cloning and/or refactoring of silent BGCs of interest for native or heterologous expression (protocols available on ActinoBase.org).

Typically, research into filamentous actinobacteria has been focused on the discovery of medicinally and industrially relevant SMs [67]. However, this is not the only reason why these organisms have been studied. These bacteria are also well known for their unique development, differentiation and complex life-cycles [68], intricate regulatory pathways [69–71], as well as their extensive ecological associations with diverse host organisms, including plants [72–74], attine ants [23, 75] and marine sponges [76]. In order to properly represent and support the diversity of research, efforts have been placed on adding subsections into the ActinoBase Wiki, wherein field-specific resources and protocols can be found on the sidebar.

**Fig. 3.**
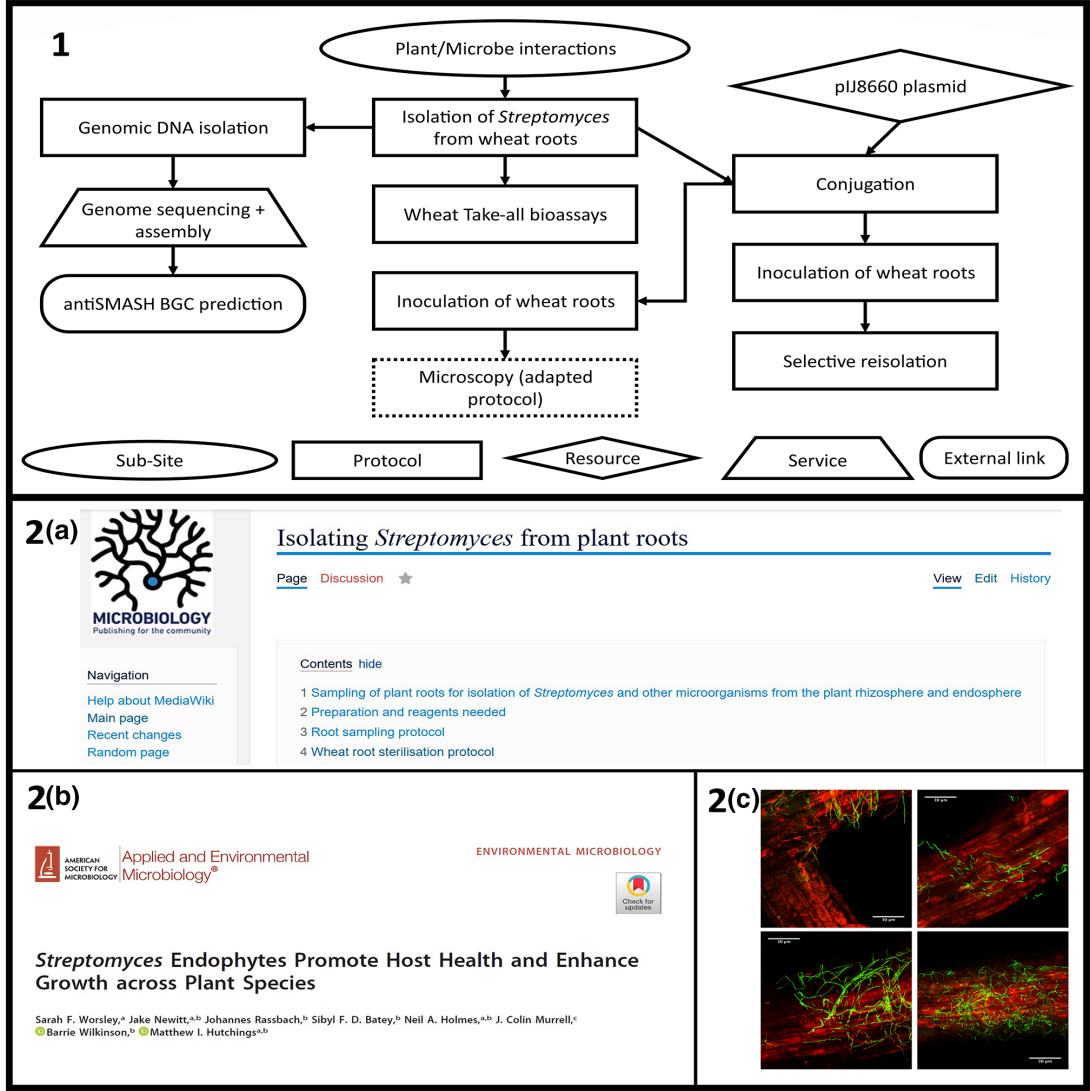
1) An example workflow for interrogating *Streptomyces *-plant root interactions using wheat. (2 a) ActinoBase provides protocols for investigating *Streptomyces *-plant interactions. These are backed up by published papers (2b), which are linked at the bottom of each page. The user can get an idea of the data that can be produced using these methods (2 c).

Field-specific subsections support the end user in the following ways: first, by summarising key research philosophies in the field; secondly, by providing specialised experimental protocols; and third, by documenting a curated list of key papers. For example, the plant-microbe interaction section focuses on the interactions of *Streptomyces* with plant roots [72]. A typical case study workflow (Fig. 3) that aims to investigate the interactions of wheat-root-associated streptomycetes and their plant host will first involve root sampling; researchers can find a specific protocol to wheat plants on the ActinoBase wiki, which has been adapted from published methods for the partially selective isolation of actinobacteria [77–80]. If the researcher wants to screen for bioactivity against agriculturally-relevant pathogens, for example against the wheat take-all fungus *Gaeumannomyces tritici,* these protocols can be found in the *in vitro* section. The *in planta* protocols section provides a workflow for wheat root inoculation and selective re-isolation, which aids the researcher in determining colonisation fitness. Further interrogating the ecology of *Streptomyces* bacteria living inside plant roots requires genetic modification of environmental isolates, with the use of an integrative vector harbouring an antibiotic resistance marker and constitutively expressing the enhanced Green Fluorescent Protein (eGFP) gene for visualisation. For this, the user is directed to relevant pages for both the conjugation protocol and the vector map. Furthermore, there is a section related to imaging GFP-tagged *Streptomyces* strains associated with the roots of *Arabidopsis thaliana*. This page displays examples of images that can be obtained, an established protocol for sample preparation and imaging, as well as a link to the publication from which the protocol was adapted. This can serve as a model for conducting similar experiments on any other plant root and, if successful, the researcher can update the Wiki with an adapted protocol for their study plant; this iterative process will ultimately diversify the protocols in this section, and help other researchers to address similar research questions.

There are two key differences that distinguish ActinoBase from other Wiki-based platforms that support scientific research: 1) ActinoBase is specifically aimed at researchers working with filamentous actinobacteria, therefore it is tailored to assist users in the challenges related to this group; 2) It provides a resource for researchers with a broad range of experience. While many researchers may have used a similar Wiki-based bioresource known as OpenWetWare (openwetware.org), that site is a general resource for protocols, and is designed to support a broader community of biologists. OpenWetWare contains some sections dedicated to *Streptomyces* biology, but ActinoBase provides a greater depth of specific information for researchers working with these microorganisms and their close relatives. The benefits of ActinoBase are multi-faceted, encouraging a tighter community of researchers. Moreover, it also provides an opportunity for non-specialised researchers in related disciplines to engage and learn more about these microbes and their associated challenges.

**Fig. 4.**
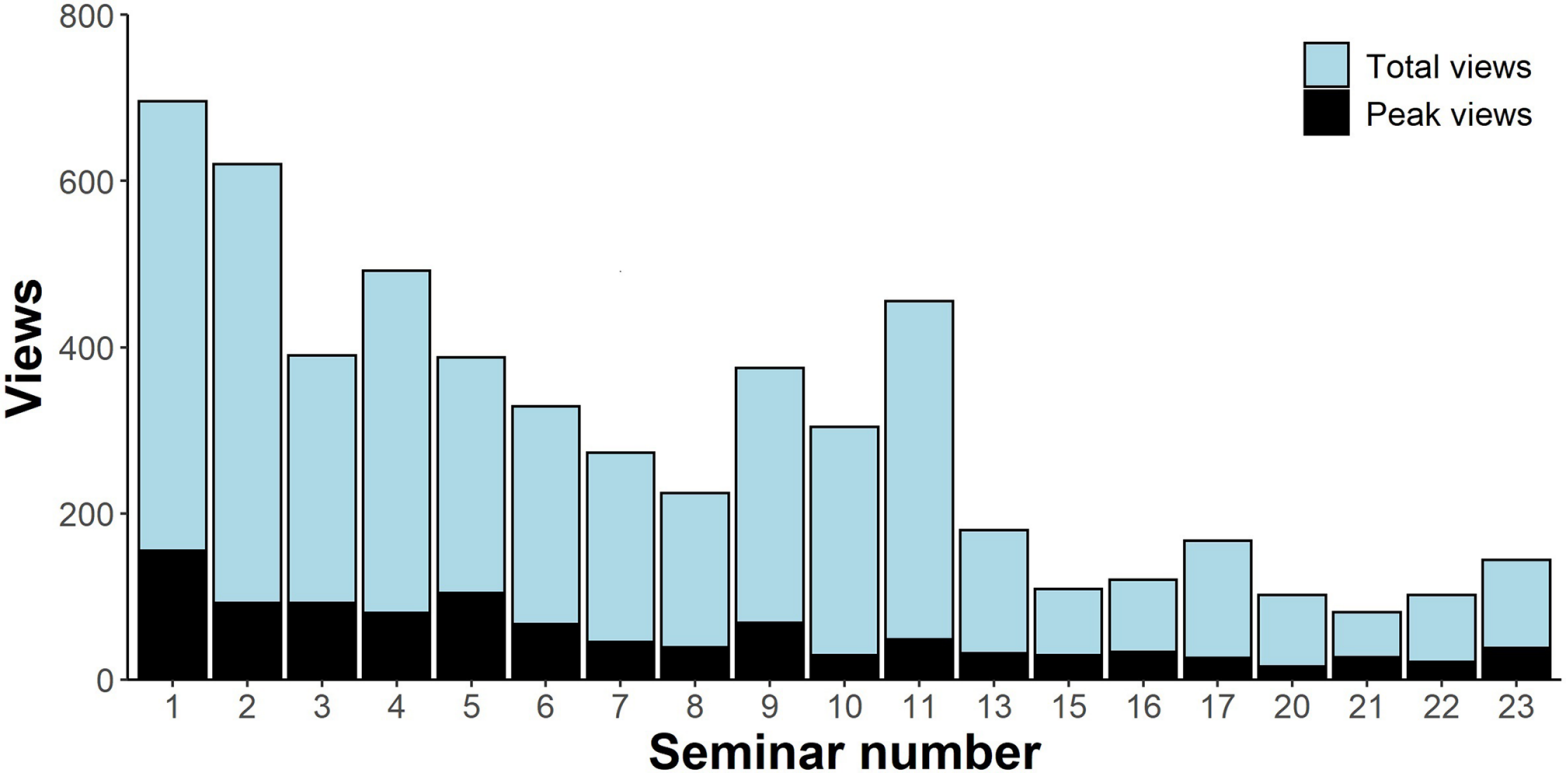
eSeminar views on the ActinoBase YouTube channel regarding both series (as of 17 November 2021). Missing eSeminars are due to private streaming, as some presenters shared unpublished results.

The impact of COVID-19, in particular on early career researchers (ECRs), has been well documented [81]. The ActinoBase eSeminars were born out of the desire to not only fill this gap, but also to provide a more inclusive and accessible framework to engage a larger, more diverse community, without the barriers of funding and travel. ActinoBase eSeminars provided a platform through which researchers could remain connected without succumbing to the limitations of the Covid-19 pandemic, and, more importantly, it allowed the community to strengthen teamwork and collaborations through online scientific presentations regardless of geographic location. Overall, a total of 13 eSeminars ran in 2020 with 20 speakers (nine PhD students, seven postdoctoral researchers and four group leaders) starting in April and ending in August. In 2021, the second series of eSeminars started in June and ended in October, and included 10 eSeminars with 11 speakers (three PhD students, three postdoctoral researchers and five group leaders) http://actinobase.org/index.php/The_ActinoBase_e-Seminar. The average number of views per seminar, as of 21 September 2021, was 394 (2020 average 503; 2021 average 130) while the total number of views in this time period was 6691 (2020 total 6041; 2021 total 650). We believe these numbers are consistent with relaxation of social restrictions as Covid-19 vaccination programmes came online globally and laboratories began operating more normally. Nevertheless, an advantage of YouTube streaming via the ActinoBase channel is that eSeminars can be recorded, allowing them to be seen not only live, but also watched later at the viewer's convenience. This permitted views to increase far beyond the number of live views, as most of the recordings are made public after live streaming, further improving the accessibility of the information presented in the talks. The impact, in terms of community creation can be illustrated by eSeminar engagement (Fig. 4).

The impact and reach of seminar engagement was broadened by sharing on social media (https://twitter.com/ActinoBase), and the creation of hashtags for questions. This activity demonstrated a supportive and dynamic community that showed an active participation in each eSeminar. This can be further evidenced by the international spread of presenters. In terms of presenters across both eSeminar series, a total of 15 countries were represented. The majority of presenters were UK-based (10 total), followed by countries like Germany, Netherlands, Canada, Denmark, United States, Mexico, Chile, Thailand, Sweden, Poland, Argentina, India, Jamaica and South Africa. Audience views were registered from the UK, United States, Mexico, India, Sweden, Germany, Denmark, Singapore, and The Netherlands.

The ActinoBase committee is mainly composed of ECRs who volunteer to curate the website and to organise the eSeminar series. ECRs receive mentoring and support from senior scientists in the field. To ensure fair representation, the committee is refreshed annually. In its second year, the committee has expanded to include ECR members from around the globe, including South Africa and Latin America. The team exchange ideas via Slack and often meet in-person during local ‘wiki-thons’ which focus on discussing, adding and editing content as well as deciding on the future direction of ActinoBase. The benefits of being involved at the committee level include: peer-to-peer networking (both locally, nationally and internationally), broadening of knowledge and skills (including Wiki editing), public acknowledgement of contributions to the Wiki (with plans to add template text which can be added to a CV) and a yearly Microbiology Society ActinoBase review publication [82, 83]. Although ActinoBase was started by members of academic research groups based in the UK, there has been a strong emphasis on expanding community involvement in order to make it more diverse, inclusive and equal. This can be evidenced by both the eSeminar series, which included presenters from underrepresented countries, and the most recent ActinoBase review publication that involved ECR collaborators from Europe, Latin America and Asia. We recognise that there are still significant improvements to be made in these areas. For instance, a future aim is to translate the Wiki into other languages predominantly spoken/used by the community members.

## Long-term use

The vision for ActinoBase is to create a ‘go to’ resource for all aspects of research into filamentous actinobacteria - everything from useful, practical (unpublished) information to a hub for curated resources. For example, genome and metabolomics databases could be integrated with ActinoBase. In addition, expanding educational resources such as lecture slide sharing, outreach activities and image storage would provide an incredible resource for the community. In the long-term, we anticipate that ActinoBase showcases community engagement and impact in relation to particular taxa or model microorganisms. In this sense, the Microbiology Society already has existing *Microbe Profiles* (microbiologyresearch.org/content/microbe-profiles), including *S. coelicolor* [84]. Incorporating these into Wikis might provide an avenue for building and strengthening existing communities through common interests and shared engagement. We believe that this Wiki could be showcased at the Microbiology Society Annual Conference as a way of bringing the community together, as well as perhaps a mentoring/shadowing element of existing curators to new members. Tying into both the Microbiology Society Annual meeting and the International Symposium on the Biology of Actinomycetes (ISBA) could provide avenues for at least yearly hybrid committee meetings. In addition, we hope that ActinoBase could provide a base template for the Microbiology Society to expand to other taxa user groups.

A key long-term focus is to expand accessibility, involvement, and engagement by prioritizing equality, diversity and inclusion amongst our content curators and knowledge users. With this in mind, we aim to survey the community about ways to get involved and ask them what they would like to see from ActinoBase in the future. This could include, for example, the creation of new Wiki pages, links to external resources, community Wikithons, educational and outreach content. We aim to raise awareness, promote the resource and encourage use and curation across the actinobacteria community through two main mechanisms, at specialised meetings such as ISBA and, to increase global engagement and inclusivity, through a series of webinars with interactive surveys. The content of these in-person and virtual events will be similar, to showcase what ActinoBase is, to gather community feedback, to widen engagement and increase participation (e.g. recruitment of curators). We also plan to broaden involvement across relevant subdisciplines (e.g. cell biology, developmental biology, bioinformatics) by creating a resource pack for dissemination – over time, we anticipate that this would include slides and infographics showcasing ActinoBase as well as video resource tutorials on how to be involved. We would aim to start with accessible content on how to upload content (curation). In this way, through broader engagement of relevant expertise, we anticipate that ActinoBase will remain current and reflective of our dynamic community, with mechanisms such as a ‘significant contributors page’ as a formal recognition of roles.

The success of ActinoBase is driven by engagement, sharing of knowledge and building of community - ultimately, we recognise this as a starting point of a long-term project. We hope it showcases the tremendous input and value of engaging ECRs, giving them space to not only lead, but also to shape the community that they wish to be a part of. In the future, we would like the impact to span decades, and inspire the next generations of researchers to collaborate globally.
